# Impact of abobotulinumtoxinA on the clinical features of cervical dystonia in routine practice

**DOI:** 10.1016/j.prdoa.2020.100063

**Published:** 2020-06-15

**Authors:** Richard M. Trosch, Vijay P. Misra, Pascal Maisonobe, Savary Om

**Affiliations:** a The Parkinson's and Movement Disorders Center, Farmington Hills, 48334, MI, USA.; b Imperial College Healthcare NHS Trust, London W12 0HS, UK.; c Ipsen, 65 Quai Georges Gorse, 92100 Boulogne-Billancourt, France

**Keywords:** Botulinum toxin, Cervical dystonia, Observational study, Meta-analysis

## Abstract

**Introduction:**

The efficacy and safety of abobotulinumtoxinA in the management of cervical dystonia has been established in randomized, controlled trials that use a selected trial population. In this meta-analysis of observational data, we evaluated the real-life effectiveness of abobotulinumtoxinA as delivered in routine clinical practice.

**Methods:**

Meta-analysis of patient-level data for adult patients with cervical dystonia treated with abobotulinumtoxinA from three prospective, multicenter, observational studies (NCT01314365, NCT00833196 and NCT01753349).

**Results:**

We report data for patients treated with abobotulinumtoxinA over one injection cycle at 181 neurology centers in 35 countries. CD clinical features as assessed by Toronto Western Spasmodic Torticollis Rating Scale (TWSTRS) Total scores (*N* = 920) significantly reduced by a mean [95%CI] of −12.9 [−13.9, −11.8] points at Week 4 (*N* = 449) and −3.2 [−3.8, −2.7] points at the end of the injection cycle (*N* = 890). All three TWSTRS domains (symptom severity, disability and pain) contributed to the overall improvement. Patients were generally content with symptom control at peak effect of the treatment cycle, with 86% reporting overall satisfaction.

**Conclusion:**

Findings from this meta-analysis of observational studies confirm the effectiveness of abobotulinumtoxinA in routine practice. Despite inclusion of a broader population sample, the magnitude of improvements observed is consistent with that seen in the pivotal, randomized controlled trials.

## Highlights

• Observational data provide insights into the effectiveness of aboBoNT-A in practice
• CD symptoms improve with aboBoNT-A and do not fully return before the next injection
• Patient satisfaction is highest when aboBoNT-A is given at recommended doses

## Introduction

Cervical dystonia (CD) is a neurological syndrome characterized by involuntary, sustained, contractions of cervical muscles causing abnormal postures of the neck [1]. It is a heterogeneous disorder, with numerous possible patterns of head and neck deviations. Additional signs and symptoms include shoulder elevation, jerking movements, neck or shoulder pain and tremor. Currently, there is no cure for CD. Available therapeutic interventions are symptomatic and are aimed at lessening the severity of the dystonic contractions and their associated symptoms. The clinical utility of oral therapies in the management of CD is limited by a narrow therapeutic window and side-effect profile [2].

Several randomized controlled trials (RCTs) [[3], [4], [5], [6], [7], [8]], and long-term studies [9] have established the efficacy and safety of botulinum neurotoxin BoNT products in the management of CD, and targeted chemodenervation with BoNT is considered the treatment of choice [7,10]. We present here a meta-analysis of patient-level data from three prospective, observational studies, with the aim of exploring the symptomatic effectiveness of abobotulinumtoxinA as given in routine clinical practice.

## Methods

The organization of the database of each of the three observational studies has previously been described [11]. In brief, the database includes patient-level data from three prospective, observational studies which followed the course of adult CD patients treated with BoNT-A [[12], [13], [14]]. In each study, the decision to treat was taken prior to, and independently from, the decision to enroll the subject in the study. For each study, Independent Ethics Committee/Institutional Review Board approval was obtained prior to each center initiation and all patients provided written informed consent.

The INTEREST IN CD studies (INTEREST IN CD1 [NCT00833196, [13]] and INTEREST IN CD2 [NCT01753349, [14,15]) were international, non-interventional studies including patients treated with any brand of botulinum neurotoxin Type A (BoNT-A). ANCHOR-CD (NCT01314365) [12] was a US non-interventional, registry study following patients treated with abobotulinumtoxinA (Dysport®, Ipsen Pharma, Wrexham, UK). Eligible patients in all three studies could be new to BoNT-A treatment or previously treated with BoNT-A, provided there had been at least a 12-week interval between the last injection and study entry. Patients enrolled in INTEREST CD1 had to have a TWSTRS severity score of ≥15 [13]. While strictly observational, all studies included comprehensive clinical CD assessments at each visit, including data on medical and treatment history, injection parameters and the Toronto Western Spasmodic Torticollis Rating Scale (TWSTRS). The INTEREST IN CD2 and ANCHOR-CD studies followed multiple injection cycles and collected data on patient satisfaction with treatment. Satisfaction with symptom control at peak effect was assessed in INTEREST IN CD2 using a 5-point Likert scale [15] and satisfaction with relief from symptoms was assessed in ANCHOR-CD using Item 2 (7-point scale) of the Treatment Satisfaction Questionnaire for Medication (TSQM) [12]. All three studies assessed patients at re-injection visits (i.e. at end of the last treatment cycle), and the INTEREST IN CD1 and ANCHOR-CD studies also included assessments at peak effect (Week 4).

### Statistical analyses

We analyzed patient level data from Cycle 1 of the observational studies. For patients enrolled in more than one study (i.e. INTEREST IN CD1 and INTEREST IN CD2 or ANCHOR-CD and INTEREST IN CD2), only data from the INTEREST IN CD2 study were retained for the meta-analysis. Due to differences in methodology, some variables were not consistently collected across the component studies. To account for differences in satisfaction assessment, satisfaction with treatment was dichotomized as ‘satisfied’ (score of 1–2 on Likert scale or 4–7 on TSQM) or ‘not satisfied’ (score of 3–5 on Likert scale including neutral, or 1–3 on TSQM). We also assessed rates of satisfaction according to 4 dose categories (≤250U, 250 - ≤500U, 500- ≤1000U, >1000U).

We present all available data for each variable. Only data from patients treated with abobotulinumtoxinA and with data collected both at baseline and the end of Cycle 1 visit from neurology clinics were considered [12]. Statistical analyses are primarily descriptive, changes in TWSTRS scores were compared versus baseline using a two-sided paired *t*-test at the 5% significance level. There was no imputation for missing data.

## Results

### Patient characteristics

These analyses include data from a total of 1091 patients with CD treated with abobotulinumtoxinA at 181 neurology centers in 35 countries. Patient demographics and medical history at baseline are presented in Table 1**.** Most patients (66%) were female and 86% were aged at least 41 years old.

**Table 1:** Demographic, medical history and clinical characteristics at baseline.

Characteristic	AbobotulinumtoxinA treated subjects (*N* = 1091)
Sex (female); n (%)	722 (66.2)
Age; n (%)	
18–30	45 (4.1)
31–40	109 (10.0)
41–50	244 (22.4)
51–60	280 (25.7)
61–70	266 (24.4)
>70	147 (13.5)
Proportion subjects with CD family history; n (%)	76 (7.0)
Time since diagnosis (years); n (%)	
<1	122 (11.2)
1–5	380 (34.8)
>5	589 (54.0)
Previous treatment with BoNT-A; n (%)	
Yes	916 (84.0)
No	174 (16.0)
Missing	1
Use of concomitant medication; n (%)	522 (47.8)
Predominant head/neck deviation pattern; n (%)	*N* = 869
Torticollis	610 (70.2)
Laterocollis	188 (21.6)
Retrocollis	40 (4.6)
Anterocollis	12 (1.4)
Lateral shift	9 (1.0)
Sagittal shift	9 (1.0)
Missing/not derived	*N* = 222

BoNT-A: botulinum neurotoxin type A; CD: cervical dystonia; SD: standard deviation.

### Injection parameters

Data for the abobotulinumtoxinA injection parameters are summarized in Table e1. The median dose of abobotulinumtoxinA was 500 U [range 50–1700 U]. Overall, less than half (39%) of patients were injected for CD using a guidance technique. The most commonly injected muscles were the splenius capitis (injected in 89% of subjects), sternocleidomastoid (80%), trapezius (62%), levator scapulae (47%), semispinalis capitis (32%) and scalene group (17%); all other relevant muscles were injected in <10% of patients.

The mean duration of the injection cycle was 111 ± 45 days (16 weeks), with a median of 99 days [estimated 75th percentile: 141 days] (i.e. median: 14 weeks, estimated 75th percentile: 20 weeks).

### CD features as assessed by the Toronto Western Spasmodic Torticollis Rating Scale

**Fig. 1:**
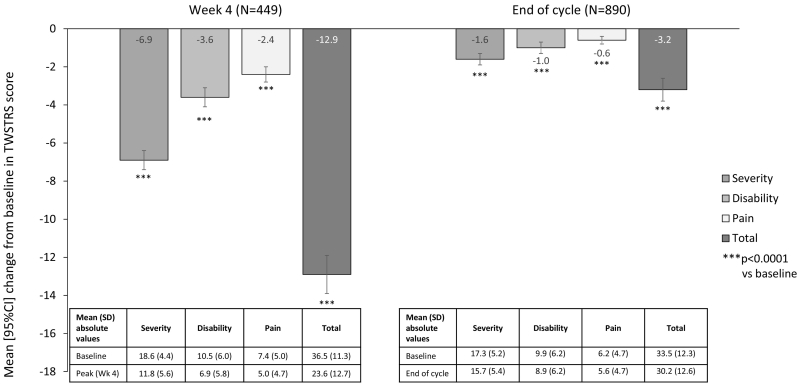
Mean change from baseline in TWSTRS scores, at peak effect (Week 4) and end of cycle. TWSTRS, Toronto Western Spasmodic Torticollis Rating Scale. Data for Week 4 are based on INTEREST IN CD1 and ANCHOR, data for end of cycle are from all three studies.

CD clinical features as assessed by TWSTRS Total scores significantly improved from baseline to Week 4, and did not return fully to baseline at end of injection cycle (*N* = 920, Fig. 1). TWSTRS Total scores significantly reduced by a mean [95%CI] of −12.9 [−13.9, −11.8] points at Week 4 (*N* = 449) and by −3.2 [−3.8, −2.7] points at the end of injection cycle (*N* = 890). All three TWSTRS domains (symptom severity, disability and pain) contributed to the overall improvement, with significant reductions versus baseline both at peak effect (Week 4) and at end of injection cycle.

### Satisfaction with CD symptom control at peak effect

Patients were generally satisfied with symptom control at peak effect of the treatment cycle, with 86% (710/826) reporting overall satisfaction.

When categorized by dose, rates of satisfaction were slightly higher when abobotulinumtoxinA was dosed per recommended dose ranges than treatment at the extremes of dosing [17]. Overall, 88% (401/465) of patients given abobotulinumtoxinA doses of 250 - ≤500 U and 86% (240/281) dosed between 500 - ≤1000 U were satisfied with their symptom control compared with 73% (51/74) given ≤250 U and 78% (18/23) given >1000 U.

## Discussion

In this meta-analysis of observational studies, treatment with abobotulinumtoxinA significantly reduced TWSTRS total scores by 12.9 points at peak effect, and patients did not fully return to baseline by the end of injection cycle. Patients were generally satisfied (86% satisfaction) with the symptom control afforded by the treatment at peak effect.

Despite the inclusion of patients with lower baseline disease severity (TWSTRS Total scores of <37 in the present sample versus >43 in the RCTs), the absolute magnitude of improvement seen in this sample is similar to that of the abobotulinumtoxinA phase 3 RCTs reported by Truong et al. in 2005 and 2010 [5,6], and more recently by Poewe et al. [8]. In those studies, the mean change from baseline to Week 4 in TWSTRS Total scores ranged from approximately −10 to −16 points versus baseline [5,6,8]. Recent analyses of the minimal clinically important change (MCIC) for TWSTRS total scores have found that mean changes of 7 to 12 points are associated with relevant change on clinician and patient global impression ratings [16,17]. The differences in proposed MCIC's reflect differences in study methodologies and anchors for change. Indeed the upper value is based on an analysis of the ANCHOR-CD cohort included in the present dataset [16]. Irrespective of which MCIC definition was used, our data confirm a meaningful effect of a single abobotulinumtoxinA injection cycle as given in routine practice. It may be of interest to assess the MCIC using outcomes such as patient satisfaction as an anchor.

The pivotal double-blind abobotulinumtoxinA studies found that significant differences in the reduction of TWSTRS scores were maintained versus placebo and baseline until Week 12 [5,6,8], and in their open-label extensions the median time to retreatment was 14 weeks [18 weeks for 75th percentile] [18]. In our sample, we observed a similarly long injection cycle duration (median of 14 weeks, 75th percentile of 20 weeks). Our findings also compare favorably with those reported in observational studies of other BoNT-A products; mean injection intervals for treatment cycle 1 were: ~14 weeks with onabotulinumtoxinA in the CD-PROBE study [19] and ~ 12 weeks in the German incobotulinumtoxinA study [20] versus a mean of ~16 weeks with abobotulinumtoxinA in the present meta-analysis. A longer duration of abobotulinumtoxinA is also supported by data from the first treatment cycle of the ULIS-III observational spasticity study, which reported a significantly longer duration of action for abobotulinumtoxinA (>30 days) between the first and second injection than patients treated with other BoNT-A products [21]. A plausible explanation for the different durations of response in both CD and spasticity is the different amounts of active neurotoxin found at the approved doses for each product [22].

There is obviously a difference in the symptom relief afforded at peak effect versus across the injection cycle. A key clinical question is how well symptoms are covered *in between* dosing sessions. A recent patient survey found that most patients treated with the different BoNT-A brands (25% receiving abobotulinumtoxinA) first noticed waning of BoNT-A effects at about 10.5 weeks with significant impact on daily activities and quality of life [23]. This suggests that many patients have to live with significant symptom re-emergence for at least a few weeks before they are reinjected. Such data has led some authors to suggest that injection sessions could be shortened to match the duration of reported efficacy for the different products [24,25], but this would clearly be off-label. Our data suggest that treatment with abobotulinumtoxinA will provide good symptom coverage of symptoms across a typical cycle. In support of this notion, a *post-hoc* analysis of the Interest in CD-2 study found that patients who attended clinics that allowed some flexibility in injection cycles (to meet individual patient needs) had longer abobotulinumtoxinA injection intervals than those who attended clinics with fixed schedules (15.3–15.9 weeks vs. 14.1 weeks, respectively), suggesting that many patients treated flexibly with abobotulinumtoxinA are able to go longer than the standard interval [26]. In our meta-analysis, most patients did not fully return to baseline before their next injection and their improvement in TWSTRS scores (−3.2 points) remained statistically significant (albeit below the MCIC). This persistence of benefit at the time of next injection suggests that patients and physicians do not wait for the full waning of effect before the next injection. Full analyses of the INTEREST IN CD2 study have thus found a gradual cumulative benefit over 3 years of treatment [15]. From clinical experience, few patients completely remit from their symptoms; however, in one naturalistic study, Skogseid et al. reported that a small number of patients could discontinue treatment because no further treatment was required [27].

A limitation of the TWSTRS is that the severity domain considers all the physical findings (symptoms) under one composite heading [28]. Without data on the scores for individual item scores, it is difficult to discern the impact of treatment on the individual symptoms of CD. By contrast, the impact of treatment on pain can be directly assessed by evaluating change in TWSTRS pain scores. This is important because some patients often assess their response to treatment based on the improvement in pain, irrespective of changes in other symptoms of CD. Patient surveys have found that the presence of pain is the main reason for patients to seek treatment [29,30] and an observational study reported that pain is often a substantial driver of disability in people living with CD. The mechanism of pain in CD remains mostly unknown, but it has been suggested to be more than just muscle related with one study reporting changes in pain threshold [31]. A recent meta-analysis found that BoNT-A can be effectively used in both muscle-based and non-muscle-based pain disorders, suggesting independent effects in pain mechanisms [32]. In this meta-analysis, we found pain significantly reduced by ≥2 points on the TWSTRS pain scale at Week 4, which equated to >30% improvement from baseline. This magnitude of improvement is consistent with the first phase 3 study reported by Truong in 2005 [6], and is slightly smaller than the reduction of >3 points recently by Poewe et al. [8]. Baseline pain scores were lower in the observational studies than in the pivotal studies, which typically include more severely affected patients (baseline pain score of 6.2 in this database versus >10.5 in the pivotal studies) [5,6,8]. Pain reductions at end of cycle (mean of 16 weeks) remained significant versus baseline (0.6 versus baseline, *p* < 0.001) but the magnitude of change was much smaller than seen at Week 12 in the RCTs, where Week 12 pain reductions were 1.7–1.8 versus baseline [5,6,8]. Other observational studies have also reported good effects of BoNT-A treatment on pain supporting pain reduction in CD as a major indicator for retreatment [33,34].

Although now recognized as an important treatment outcome, satisfaction with treatment has not often been assessed in prior RCTs. Studies have shown that satisfaction with symptom control is highest at peak symptomatic effect and lowest at end of cycle when the therapeutic response has waned [15,24]. Factors such as effective pain relief and the overall improvements all TWSTRS domains likely account for the high ratings of patient satisfaction with symptom control at peak effect. Subgroup analyses found that more patients treated with abobotulinumtoxinA at recommended dose ranges for CD [18] were more satisfied with symptom control than those treated with low (<250 U) and high (>1000 U) doses. At lower than recommended doses, the lower rates of satisfaction may reflect a subtherapeutic dose or a restricted number of injected muscles. Indeed, the lowest recorded dose was 50 U (which is mostly below the recommended minimum dose for the top 5 injected muscles) and some patients received injections into only one muscle. On the other hand, dissatisfaction with higher doses may reflect factors such as adverse events or a higher baseline severity that necessitated use of higher doses that went as high as 1700 U in this meta-analysis of routine practice studies. Primary analyses from the INTEREST IN CD2 study found that worse disease severity at baseline is predictive of lower treatment satisfaction at end of treatment cycle [15]. While dosing must be tailored to the individual, evidence-based recommendations for dose ranges are available per muscle for abobotulinumtoxinA and other BoNT-A products [35].

A key strength of these analyses is the size of population included. The data presented herein represent the largest dataset of subjects with CD studied to date. The observational studies included had international reach, thereby improving the generalizability of data. However, we cannot rule out the potentially confounding influence of including data from countries with very different access to services and treatment. We limited our analyses to abobotulinumtoxinA because most subjects across the trials were treated with this formulation (ANCHOR-CD study was restricted to abobotulinumtoxinA) and for comparability with the pivotal studies. Outcomes were assessed based on all available data. For example, whereas assessment of TWSTRS at Week 4 was based on data from INTEREST IN CD1 and ANCHOR-CD, assessment at end of treatment cycle was based on INTEREST IN CD1 and INTEREST IN CD2. Other limitations include those inherent to observational studies (e.g. level of missing data), as well as the recruitment of smaller subject numbers in many countries and potential site selection bias.

The finding of this meta-analysis of 3 observational studies confirms the effectiveness of abobotulinumtoxinA as used in routine practice. Despite inclusion of a broader population sample, the magnitude of improvements seen was consistent with those seen in prior level 1 RCTs.

The following is the supplementary data related to this article.

Table e1 Administration of abobotulinumtoxinA Table e1

## CRediT authorship contribution statement

**Richard M. Trosch:**Conceptualization, Investigation, Methodology, Writing - original draft.**Vijay P. Misra:**Conceptualization, Investigation, Methodology, Writing - review & editing.**Pascal Maisonobe:**Formal analysis, Software, Writing - review & editing.**Savary Om:**Conceptualization, Methodology, Project administration, Writing - review & editing.

## References

[1] Albanese A., Bhatia K., Bressman S.B., Delong M.R., Fahn S., Fung V.S., Hallett M., Jankovic J., Jinnah H.A., Klein C., Lang A.E., Mink J.W., Teller J.K. (2013). Phenomenology and classification of dystonia: a consensus update. Mov. Disord..

[2] Jankovic J. (2006). Treatment of dystonia. Lancet Neurol..

[3] Poewe W., Deuschl G., Nebe A., Feifel E., Wissel J., Benecke R., Kessler K.R., Ceballos-Baumann A.O., Ohly A., Oertel W., Kunig G. (1998). What is the optimal dose of botulinum toxin A in the treatment of cervical dystonia? Results of a double blind, placebo controlled, dose ranging study using Dysport German Dystonia Study Group. J. Neurol. Neurosurg. Psychiatry.

[4] Wissel J., Kanovsky P., Ruzicka E., Bares M., Hortova H., Streitova H., Jech R., Roth J., Brenneis C., Muller J., Schnider P., Auff E., Richardson A., Poewe W. (2001). Efficacy and safety of a standardised 500 unit dose of Dysport (clostridium botulinum toxin type A haemaglutinin complex) in a heterogeneous cervical dystonia population: results of a prospective, multicentre, randomised, double-blind, placebo-controlled, parallel group study. J. Neurol..

[5] Truong D., Brodsky M., Lew M., Brashear A., Jankovic J., Molho E., Orlova O., Timerbaeva S., Global Dysport Cervical Dystonia Study G (2010). Long-term efficacy and safety of botulinum toxin type A (Dysport) in cervical dystonia. Parkinsonism Relat. Disord..

[6] Truong D., Duane D.D., Jankovic J., Singer C., Seeberger L.C., Comella C.L., Lew M.F., Rodnitzky R.L., Danisi F.O., Sutton J.P., Charles P.D., Hauser R.A., Sheean G.L. (2005). Efficacy and safety of botulinum type A toxin (Dysport) in cervical dystonia: results of the first US randomized, double-blind, placebo-controlled study. Mov. Disord..

[7] Simpson D.M., Hallett M., Ashman E.J. (2016). Practice guideline update summary: Botulinum neurotoxin for the treatment of blepharospasm, cervical dystonia, adult spasticity, and headache. Neurology.

[8] Poewe W., Burbaud P., Castelnovo G., Jost W.H., Ceballos-Baumann A.O., Banach M., Potulska-Chromik A., Ferreira J.J., Bihari K., Ehler E., Bares M., Dzyak L.A., Belova A.N., Pham E., Liu W.J., Picaut P. (2016). Efficacy and safety of abobotulinumtoxinA liquid formulation in cervical dystonia: a randomized-controlled trial. Mov. Disord..

[9] Colosimo C., Tiple D., Berardelli A. (2012). Efficacy and safety of long-term botulinum toxin treatment in craniocervical dystonia: a systematic review. Neurotox. Res..

[10] Albanese A., Asmus F., Bhatia K.P., Elia A.E., Elibol B., Filippini G., Gasser T., Krauss J.K., Nardocci N., Newton A., Valls-Sole J. (2011). EFNS guidelines on diagnosis and treatment of primary dystonias. Eur. J. Neurol..

[11] Misra V.P., Trosch R.M., Maisonobe P., Om S. (2018). Spectrum of practice in the routine management of cervical dystonia with abobotulinumtoxinA: findings from three prospective open-label observational studies. J Clin Mov Disord.

[12] Trosch R.M., Espay A.J., Truong D., Gil R., Singer C., LeWitt P.A., Lew M.F., Tagliati M., Adler C.H., Chen J.J., Marchese D., Comella C.L. (2017). Multicenter observational study of abobotulinumtoxinA neurotoxin in cervical dystonia: the ANCHOR-CD registry. J. Neurol. Sci..

[13] Misra V.P., Ehler E., Zakine B., Maisonobe P., Simonetta-Moreau M. (2012). Factors influencing response to Botulinum toxin type A in patients with idiopathic cervical dystonia: results from an international observational study. BMJ Open.

[14] Misra V.P., Colosimo C., Charles D., Chung T.M., Maisonobe P., Om S., group IICs (2018). INTEREST IN CD2, a global patient-centred study of long-term cervical dystonia treatment with botulinum toxin. J. Neurol..

[15] Colosimo C., Charles D., Misra V.P., Maisonobe P., Om S., group IICs (2019). How satisfied are cervical dystonia patients after 3 years of botulinum toxin type A treatment? Results from a prospective, long-term observational study. J. Neurol..

[16] Espay A.J., Trosch R., Suarez G., Johnson J., Marchese D., Comella C. (2018). Minimal clinically important change in the Toronto Western Spasmodic Torticollis Rating Scale. Parkinsonism Relat. Disord..

[17] Dashtipour K., Mari Z., Jankovic J., Adler C.H., Schwartz M., Brin M.F. (2019). Minimal clinically important change in patients with cervical dystonia: results from the CD PROBE study. J. Neurol. Sci..

[18] Dysport (abobotulinumtoxinA) for Injection, for Intramuscular Use. Full US Prescribing Information 2019. Available at http://dysport.com. Last accessed May 2020.

[19] Jankovic J., Adler C.H., Charles D., Comella C., Stacy M., Schwartz M., Manack Adams A., Brin M.F. (2015). Primary results from the cervical dystonia patient registry for observation of onabotulinumtoxina efficacy (CD PROBE). J. Neurol. Sci..

[20] Dressler D., Paus S., Seitzinger A., Gebhardt B., Kupsch A. (2013). Long-term efficacy and safety of incobotulinumtoxinA injections in patients with cervical dystonia. J. Neurol. Neurosurg. Psychiatry.

[21] Turner-Stokes L., Ashford S., Fheodoroff K., Brashear A., Maisonobe P., Lysandropoulos A., Jacinto J. (2019). Time to retreatment with botulinum toxin A in upper limb spasticity management: upper limb international spasticity (ULIS)-III study interim analysis. Toxicon.

[22] Field M., Splevins A., Picaut P., van der Schans M., Langenberg J., Noort D., Foster K. (2018). AbobotulinumtoxinA (Dysport), OnabotulinumtoxinA (Botox), and IncobotulinumtoxinA (Xeomin) neurotoxin content and potential implications for duration of response in patients. Toxins (Basel).

[23] Ferreira J.J., Comella C., Azoulai M., Pain E., Om S. (2020). How do patients with cervical dystonia (CD) experience their botulinum neurotoxinA (BoNT-A) treatment cycle: results from an international online survey. Eur. J. Neurol..

[24] Sethi K.D., Rodriguez R., Olayinka B. (2012). Satisfaction with botulinum toxin treatment: a cross-sectional survey of patients with cervical dystonia. J. Med. Econ..

[25] Wissel J. (2018). Towards flexible and tailored botulinum neurotoxin dosing regimens for focal dystonia and spasticity - insights from recent studies. Toxicon.

[26] Misra V.P., Charles D., Colosimo C., Chung T.M., Maisonobe P., Om S. (2020). Impact of injection schedule flexibility on patient satisfaction with botulinum toxin treatment for cervical dystonia. Neurology.

[27] Skogseid I.M., Kerty E. (2005). The course of cervical dystonia and patient satisfaction with long-term botulinum toxin A treatment. Eur. J. Neurol..

[28] Jost W.H., Hefter H., Stenner A., Reichel G. (2013). Rating scales for cervical dystonia: a critical evaluation of tools for outcome assessment of botulinum toxin therapy. J. Neural Transm..

[29] Novak I., Campbell L., Boyce M., Fung V.S. (2010). Botulinum toxin assessment, intervention and aftercare for cervical dystonia and other causes of hypertonia of the neck: international consensus statement. Eur. J. Neurol..

[30] Camargo C.H., Cattai L., Teive H.A. (2015). Pain relief in cervical dystonia with botulinum toxin treatment. Toxins.

[31] Lobbezoo F., Tanguay R., Thon M.T., Lavigne G.J. (1996). Pain perception in idiopathic cervical dystonia (spasmodic torticollis). Pain.

[32] Siongco P.R.L., Rosales R.L., Moore A.P., Freynhagen R., Arimura K., Kanovsky P., Kaji R., Fernandez H.H., Dressler D. (2020). Botulinum neurotoxin injections for muscle-based (dystonia and spasticity) and non-muscle-based (neuropathic pain) pain disorders: a meta-analytic study. J. Neural Transm..

[33] Charles P.D., Manack Adams A., Davis T., Bradley K., Schwartz M., Brin M.F., Patel A.T. (2016). Neck pain and cervical dystonia: treatment outcomes from CD PROBE (cervical dystonia patient registry for observation of OnabotulinumtoxinA efficacy). Pain Pract.

[34] Fernandez H.H., Pagan F., Danisi F., Greeley D., Jankovic J., Verma A., Sethi K., Pappert E.J., Group XCS (2013). Prospective study evaluating IncobotulinumtoxinA for cervical dystonia or blepharospasm: interim results from the first 145 subjects with cervical dystonia. Tremor Other Hyperkinet Mov (N Y).

[35] Albanese A, Abbruzzese G, Dressler D, Duzynski W, Khatkova S, Marti MJ, Mir P, Montecucco C, Moro E, Pinter M, Relja M, Roze E, Skogseid IM, Timerbaeva S, Tzoulis C (2015) Practical guidance for CD management involving treatment of botulinum toxin: a consensus statement. J. Neurol. 262**,** 3301–2213. 10.1007/s00415-015-7703-x PMC4608989 25877834

