# Supplementary material for: Impact of abobotulinumtoxinA on the clinical features of cervical dystonia in routine practice
Source: Clin Park Relat Disord. 2020 Jun 15;3:100063. doi: 10.1016/j.prdoa.2020.100063 (PMC8298811; doi:10.1016/j.prdoa.2020.100063)
Supplement: Table e1 — Administration of abobotulinumtoxinA [file mmc1.docx]

**Supplementary Appendix**

**Table e1. Administration of abobotulinumtoxinA**

| **Parameter** | **Overall** |
| --- | --- |
| **Administration at baseline (Visit 1)** | **(N=843)*** |
| Total aboBoNT-A dose (U);  mean ±SD  median [range] | 552 ± 247  500 [50–1700] |
| Number of muscles injected; median [range] | 3 [1–10] |
| Most commonly injected muscles; n (% patients)  Splenius capitis  Sternocleidomastoid  Trapezius  Levator scapulae  Semispinalis capitis  Scalene group**  Longissimus group**  Splenius cervicis  Obliquus capitis | 752 (89.2)  672 (79.7)  523 (62.0)  393 (46.6)  272 (32.3)  139 (16.5)  70 (8.3)  49 (5.8)  40 (4.7) |
| Number of injection points; median [range] | 7.0 [1–34] |
| Injected volume (mL); median [range] | 2.00 [0.2–12.0] |
| Use of injection guidance; n (%) | 329 (39.0) |
| **Injection interval (Visit 1 to Visit 2)***** | **N=1091** |
| Mean ±SD  Median [range]  Categories  <12 weeks  12-16 weeks  >16 weeks  Missing | 111.0 ± 45.2  99.0 (43–582)  32 (3.0)  793 (73.9)  248 (23.1)  18 |

** Missing data for one subject. **Scalene group includes muscles reported in the eCRF as scalenus or scalene (medius, anterior and/or posterior). Longissimus group includes muscles reported in the eCRF as longissimus, longissimus capitis and/or longissimus cervicis. AboBoNT-A: abobotulinumtoxinA; mL: milliliter; SD: standard deviation; U: units. ***Visit 1=baseline; Visit 2=end of treatment cycle 1 (next injection visit).*
